# Towards a Unified Set of Diagnostic Criteria for Multiple Sclerosis

**DOI:** 10.1002/ana.27145

**Published:** 2024-11-28

**Authors:** Wallace J. Brownlee, Angela Vidal‐Jordana, Madiha Shatila, Eva Strijbis, Lisa Schoof, Joep Killestein, Frederik Barkhof, Luca Bollo, Alex Rovira, Jaume Sastre‐Garriga, Mar Tintore, Maria A. Rocca, Federica Esposito, Matteo Azzimonti, Massimo Filippi, Benedetta Bodini, Andrea Lazzarotto, Bruno Stankoff, Xavier Montalban, Ahmed T. Toosy, Alan J. Thompson, Olga Ciccarelli, for the MAGNIMS Study Group

**Affiliations:** ^1^ Queen Square Multiple Sclerosis Center, Department of Neuroinflammation UCL Institute of Neurology London UK; ^2^ NIHR University College London Hospitals Biomedical Research Center London UK; ^3^ Department of Neurology and Multiple Sclerosis Center of Catalonia (Cemcat), Vall d'Hebron University Hospital Universitat Autònoma de Barcelona (UAB) Barcelona Spain; ^4^ Multiple Sclerosis Center Amsterdam, Vrije Universiteit Amsterdam, Amsterdam Neuroscience Amsterdam University Medical College VUMC Amsterdam the Netherlands; ^5^ Center for Medical Image Computing University College London London UK; ^6^ Section of Neuroradiology. Department of Radiology, Vall d'Hebron University Hospital Universitat Autònoma de Barcelona (UAB) Barcelona Spain; ^7^ Universitat de Vic‐Universitat Central de Catalunya Barcelona Spain; ^8^ Neuroimaging Research Unit, Institute of Experimental Neurology, Division of Neuroscience IRCCS San Raffaele Scientific Institute Milan Italy; ^9^ Neurology Unit IRCCS San Raffaele Scientific Institute Milan Italy; ^10^ Vita‐Salute San Raffaele University Milan Italy; ^11^ Paris Brain Institute Sorbonne Université Paris France; ^12^ AP‐HP Hôpital Universitaire Pitié‐Salpêtrière Paris France

## Abstract

**Objective:**

The 2017 McDonald criteria continued the separation of diagnostic criteria for relapsing–remitting multiple sclerosis (RRMS) and primary progressive MS (PPMS) for historical, rather than biological, reasons. We aimed to explore the feasibility of a single, unified set of diagnostic criteria when applied to patients with suspected PPMS.

**Methods:**

We retrospectively identified patients evaluated for suspected PPMS at 5 European centers. The 2017 McDonald PPMS criteria was the gold standard against which the 2017 McDonald RRMS dissemination in space (DIS) and dissemination in time criteria were evaluated. We also investigated modified RRMS DIS criteria, including: (i) optic nerve lesions; (ii) ≥2 spinal cord lesions; and (iii) higher fulfilment of DIS criteria alone (lesions in ≥3 regions) without dissemination in time/positive cerebrospinal fluid, for a diagnosis of PPMS.

**Results:**

A total of 282 patients were diagnosed with PPMS using the 2017 McDonald criteria, and 40 with alternate disorders. The 2017 McDonald RRMS DIS criteria and the modified DIS criteria including the optic nerve or ≥2 spinal cord lesions performed well in PPMS diagnosis when combined with dissemination in time/positive cerebrospinal fluid (sensitivity 92.9–95.4%, specificity 95%, accuracy 93.2–95.3%). A diagnosis of PPMS based on high fulfillment of modified RRMS DIS criteria had high specificity, but low sensitivity. A diagnostic algorithm applicable to patients evaluated for suspected MS is proposed.

**Interpretation:**

The 2017 McDonald RRMS criteria and modifications to DIS criteria, currently under consideration, performed well in PPMS diagnosis. Forthcoming revisions to the McDonald criteria should consider a single, unified set of diagnostic criteria for MS. ANN NEUROL 2025;97:571–582

Approximately 10–15% of people with multiple sclerosis (MS) present with slowly worsening symptoms from onset, referred to as primary progressive MS (PPMS).^1^ Initial PPMS diagnostic criteria were proposed in 2000, requiring 12 months of progressive worsening, cerebrospinal fluid (CSF)‐specific oligoclonal bands (OCBs), and dissemination in space (DIS) on magnetic resonance imaging (MRI).^2^ This approach to PPMS diagnosis has been retained in the McDonald criteria,^3^, ^4^, ^5^, ^6^ and diagnostic criteria for PPMS remain distinct from relapsing–remitting MS (RRMS). A diagnosis of PPMS, according to the 2017 McDonald criteria, can be made in patients with at least 12 months of disability progression plus 2 of: (1) ≥1 T2‐hyperintense lesion in 1 typical brain region: periventricular, cortical/juxtacortical, or infratentorial; (2) ≥2 T2‐hyperintense spinal cord lesions; and (3) CSF‐specific OCBs, after excluding other disorders.

The rationale for separate PPMS diagnostic criteria relates to the unique presentation, and wide differential diagnosis for progressive neurological syndromes.^2^, ^7^, ^8^ However, the need for separate PPMS diagnostic criteria in contemporary practice is unclear. There is increasing consensus that the pathophysiology of RRMS and PPMS share greater similarities than differences.^9^ Acute inflammatory demyelinating lesions on MRI (manifesting clinically as acute relapses) occur in PPMS,^10^, ^11^ and neurodegeneration, the main determinant of clinical progression, reflected by brain atrophy, begins early in RRMS.^12^ There are calls for a disease classification linked to biological mechanisms that considers MS as a continuum with inflammation and neurodegeneration present at disease onset in all MS patients.^9^ Revisions to the disease course classification are expected soon, and will incorporate shared underlying biology between relapsing and progressive MS.^13^ The absence of imaging or body fluid biomarkers that differentiate PPMS from RRMS^14^ further weakens arguments for separate diagnostic criteria. Finally, unified criteria will simplify the diagnostic process, increasing their applicability and ease of use in clinical settings, facilitating earlier treatment.

In the present study, we investigate the feasibility of a single, unified set of diagnostic criteria for MS, irrespective of whether the initial clinical presentation is with a relapse or insidious neurological progression. We evaluated potential modifications to RRMS DIS to test whether the same criteria can be applied to patients with progression at onset, thereby informing the forthcoming revisions to MS diagnostic criteria. We addressed 5 key questions:

(1) What is the diagnostic performance (sensitivity, specificity, accuracy, positive predictive value, and negative predictive value) of the 2017 McDonald RRMS DIS and dissemination in time (DIT) criteria, when they are applied to patients with suspected PPMS instead of patients with clinically isolated syndrome?

(2) Can the optic nerve serve as a fifth region in RRMS DIS criteria to diagnose PPMS?

(3) Can ≥2 spinal cord lesions provide an alternative MRI criterion in RRMS DIS criteria (including in patients with normal brain MRI) to diagnose PPMS?

(4) What is the performance of RRMS DIS criteria requiring ≥3 periventricular lesions in patients with suspected PPMS?

(5) Can PPMS be diagnosed using standard RRMS DIS on MRI alone, or modified DIS including the optic nerve, without positive CSF/DIT‐MRI?

## Methods

### Participants

The present retrospective study was run within the MAGNIMS network (www.magnims.eu). Centers were asked to identify patients meeting the following inclusion criteria: (1) >18 years evaluated for suspected PPMS; (2) symptom duration <10 years at the time of initial evaluation; (3) brain MRI with T2‐weighted/proton density‐weighted/fluid‐attenuated inversion recovery sequences, with or without contrast‐enhanced T1‐weighted sequences; (4) whole spinal cord MRI with T2‐weighted/proton density‐weighted/short tau inversion recovery sequences, with or without contrast‐enhanced T1‐weighted sequences; (5) CSF examination, or a follow‐up MRI for DIT; and (6) ≥24 months of clinical follow up. Centers were asked to retrospectively identify any patient evaluated for PPMS from local databases, outpatient records, or inpatient consultations, including people ultimately diagnosed with other disorders.

The final study protocol was approved by the European MAGNIMS collaboration, and ethical approval was obtained at each participating center. Data were shared in accordance with a MAGNIMS data transfer agreement.

### Data Collection

Anonymized electronic case report forms were collected. Each center was asked to enter: demographic/clinical characteristics; final diagnosis made by the local neurologist (PPMS or alternative diagnosis); number and location of T2‐hyperintense lesions in the first brain and spinal cord MRI scan acquired as part of the first diagnostic assessment (or baseline), and new T2‐hyperintense lesions at the first available follow up; the presence of gadolinium‐enhancing lesions (when available); OCB status in patients with CSF data; results of visual evoked potential (VEP) testing (when available). The term “baseline” is used to indicate the MRI and other paraclinical tests performed in the initial diagnostic work‐up, and “follow up” refers to the first available MRI used to look for new T2 and/or gadolinium‐enhancing lesions. Study data were obtained from electronic health record or local registries, with investigations performed as part of routine clinical care. The case report form was checked by a single neurologist, who confirmed the diagnosis of 2017 McDonald PPMS.

### Addressing the 5 Questions

Question 1: we tested the performance of the 2017 McDonald RRMS DIS criteria (≥1 T2‐hyperintense lesion in ≥2 of 4 regions: periventricular, cortical/juxtacortical, infratentorial, spinal cord) alone, then in combination with CSF‐specific OCBs and CSF‐specific OCBs, and/or DIT‐MRI (gadolinium‐enhancing and non‐enhancing lesions, new T2‐hyperintense lesion at follow‐up) for a diagnosis of PPMS.

Question 2: we tested the performance of modified RRMS DIS criteria, requiring lesions in ≥2 of 5 regions including the optic nerve, alone and in combination with CSF‐specific OCBs, or CSF‐specific OCBs/DIT‐MRI for a diagnosis of PPMS.

Question 3: we tested the performance of ≥2 spinal cord lesions for a diagnosis of PPMS. We then examined the 2017 RRMS DIS MRI criteria or ≥2 spinal cord lesions (ie, patients not fulfilling the 2017 DIS criteria were then tested for the presence of ≥2 spinal cord lesions, including patients with normal brain MRI) alone and in combination with CSF‐specific OCBs, or CSF‐specific OCBs/DIT‐MRI for a diagnosis of PPMS.

Question 4: we tested modified DIS MRI criteria ≥3 lesions to confirm involvement of the periventricular region (rather than ≥1 lesion), alone and in combination with CSF‐specific OCBs, or CSF‐specific OCBs/DIT‐MRI for a diagnosis of PPMS.

Question 5: we tested criteria requiring more stringent fulfillment of DIS on MRI with lesions in ≥3 of 4 regions, or ≥3 of 5 regions including the optic nerve, for a diagnosis of PPMS, without CSF‐specific OCBs/DIT‐MRI for a diagnosis of PPMS.

### Statistical Analysis

Statistical analyses were carried out using SPSS‐21 (IBM, Armonk, NY, USA). Descriptive statistics were performed on the baseline and follow‐up variables. Normally distributed continuous variables were summarized using means and standard deviations. Otherwise, continuous variables were summarized using medians and ranges, and categorical variables as percentages.

The performance of each set of criteria for a diagnosis of 2017 McDonald PPMS was estimated by calculating the sensitivity, specificity, accuracy, positive predictive value (PPV), and negative predictive value, with 95% confidence intervals (CIs). We conducted all analyses in two overlapping cohorts. First, we analyzed patients who had a CSF examination, then we analyzed the whole cohort, irrespective of whether a CSF examination was carried out or not.

## Results

**FIGURE 1:**
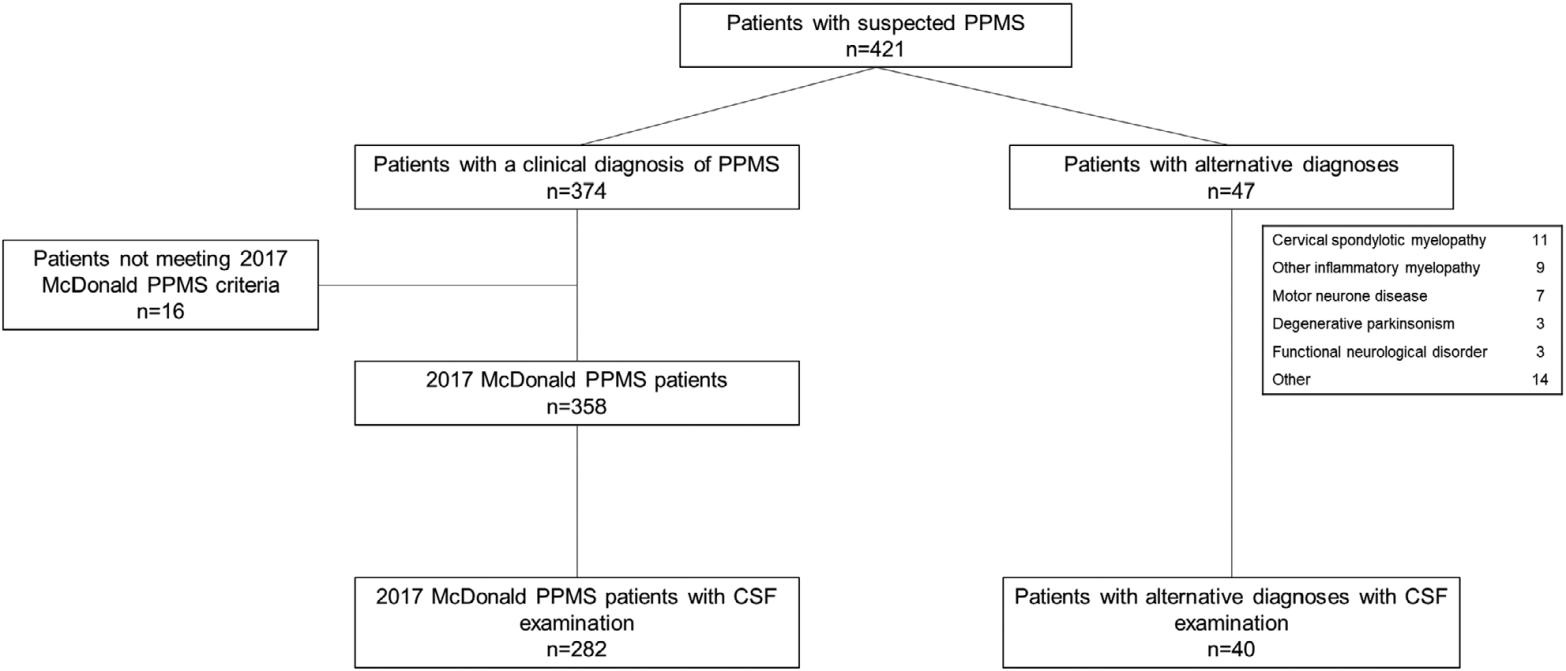
Flow chart showing patient disposition. CSF = cerebrospinal fluid; PPMS = primary progressive multiple sclerosis.

A total of 421 suspected PPMS patients, meeting the inclusion criteria, were identified from 5 MAGNIMS centers (London n = 182, Amsterdam n = 83, Barcelona n = 76, Milan n = 55, Paris n = 25). The patients were evaluated between 1996 and 2022, with a mean duration of follow up of 8.2 years (SD 5.6 years). In total, 358 patients had a confirmed diagnosis of PPMS using the 2017 McDonald criteria, and 47 were diagnosed with alternative disorders (Figure 1). A further 16 patients had a clinical diagnosis of PPMS, but did not satisfy the 2017 McDonald criteria and were excluded. Of the remaining 405 patients, 322 underwent CSF examination (282 with PPMS, 40 with alternative diagnoses) and were used in the primary analysis. Data from all 405 patients who had either a CSF examination or a follow‐up MRI scan were used in secondary analyses.

### Characteristics of Patients Evaluated for PPMS

The demographic profile was typical of early PPMS (mean age 48.8 years [range 18–78 years], women 47.1%). At the time of initial evaluation, the mean disease duration was 3.2 years (SD 2.3 years). The initial clinical presentations included progressive myelopathy (87%), progressive cerebellar ataxia (8%), and other less common syndromes (5%), such as progressive hemiparesis and multifocal presentations. The findings from the initial clinical evaluation are presented in Table 1. The baseline brain MRI scans were abnormal in 274 (97.2%) PPMS patients; all 8 (2.8%) patients with a normal brain MRI had ≥2 spinal cord lesions and positive CSF. Baseline spinal cord MRI scans were abnormal in 262 (92.9%) PPMS patients; 216 (76.6%) patients had ≥2 spinal cord lesions. CSF‐specific OCBs were found in 260 (92.2%) PPMS patients, and VEPs were abnormal in 53 of 90 (58.9%) patients tested (Table 1). The baseline demographic and clinical profile was similar in younger (aged <50 years) and older (aged >50 years) patients with early PPMS, with the exception of gadolinium‐enhancing lesions, which were more common in patients aged <50 years in the baseline brain (16.5 vs 6.1%) and spinal cord (11.6 vs 4.9%) MRI scans compared with patients aged >50 years (Supplementary Table 1).

**TABLE 1:** Baseline Characteristics of Patients with Primary Progressive Multiple Sclerosis According to the 2017 McDonald Diagnostic Criteria and Alternate Disorders

	PPMS patients according to 2017 McDonald criteria (n = 282)	PPMS mimics (n = 40)
Mean age, years (SD)	48.8 (11.5)	55.8 (9.9)
Sex (F), n (%)	133 (47.1)	19 (47.5)
Clinical presentation, n (%)		
Myelopathy	246 (87.2)	26 (66.0)
Brainstem/cerebellar syndrome	22 (7.8)	8 (20.0)
Other syndromes^a^	14 (5.0)	6 (14.0)
Progression over >12 months, n (%)	259 (91.8)	28 (70.0)
Brain MRI findings, n (%)		
Normal^b^	8 (2.8)	21 (52.5)
≥1 Periventricular lesions	261 (92.6)	17 (42.5)
≥1 Cortical/juxtacortical lesions	202 (71.6)	2 (5.0)
≥1 Infratentorial lesions	178 (63.1)	6 (15.0)
≥1 Gadolinium‐enhancing lesions at baseline^c^	24/197 (12.2)	1/30 (3.3)
≥1 New lesion at follow up^d^	73/221 (33.0)	1/28 (3.6)
Spinal cord MRI findings, n (%)		
Normal	20 (7.1)	29 (72.5)
1 Spinal cord lesion	46 (16.3)	9 (22.5)
≥2 Spinal cord lesions	216 (76.6)	2 (5.0)
≥1 Gadolinium‐enhancing lesions at baseline^c^	14/154 (9.1)	3/30 (10.0)
≥1 New lesion at follow‐up^d^	27/133 (20.3)	1/10 (10.0)
CSF‐specific oligoclonal bands, n (%)	260 (92.2)	9 (22.5)
Abnormal VEPs, n (%)^e^	53/90 (58.9)	2/20 (10.0)

All patients had a cerebrospinal fluid (CSF) examination.

PPMS = primary progressive multiple sclerosis.

^a^ Other presentations for example progressive hemiparesis, multifocal symptoms.

^b^ Patients without T2 hyperintense lesions in the periventricular, cortical/juxtacortical or infratentorial regions.

^c^ Over the number of patients who had a post‐contrast T1 scan.

^d^ Over the number of patients who had a follow‐up magnetic resonance imaging (MRI) scan.

^e^ Over the number of patients with visual evoked potential (VEP) information.

A total of 18 alternative diagnoses were made in 40 patients, most often cervical spondylotic myelopathy, other inflammatory myelopathies, and motor neuron disease (Supplementary Table 2).

### Diagnostic Criteria Performance

**FIGURE 2:**
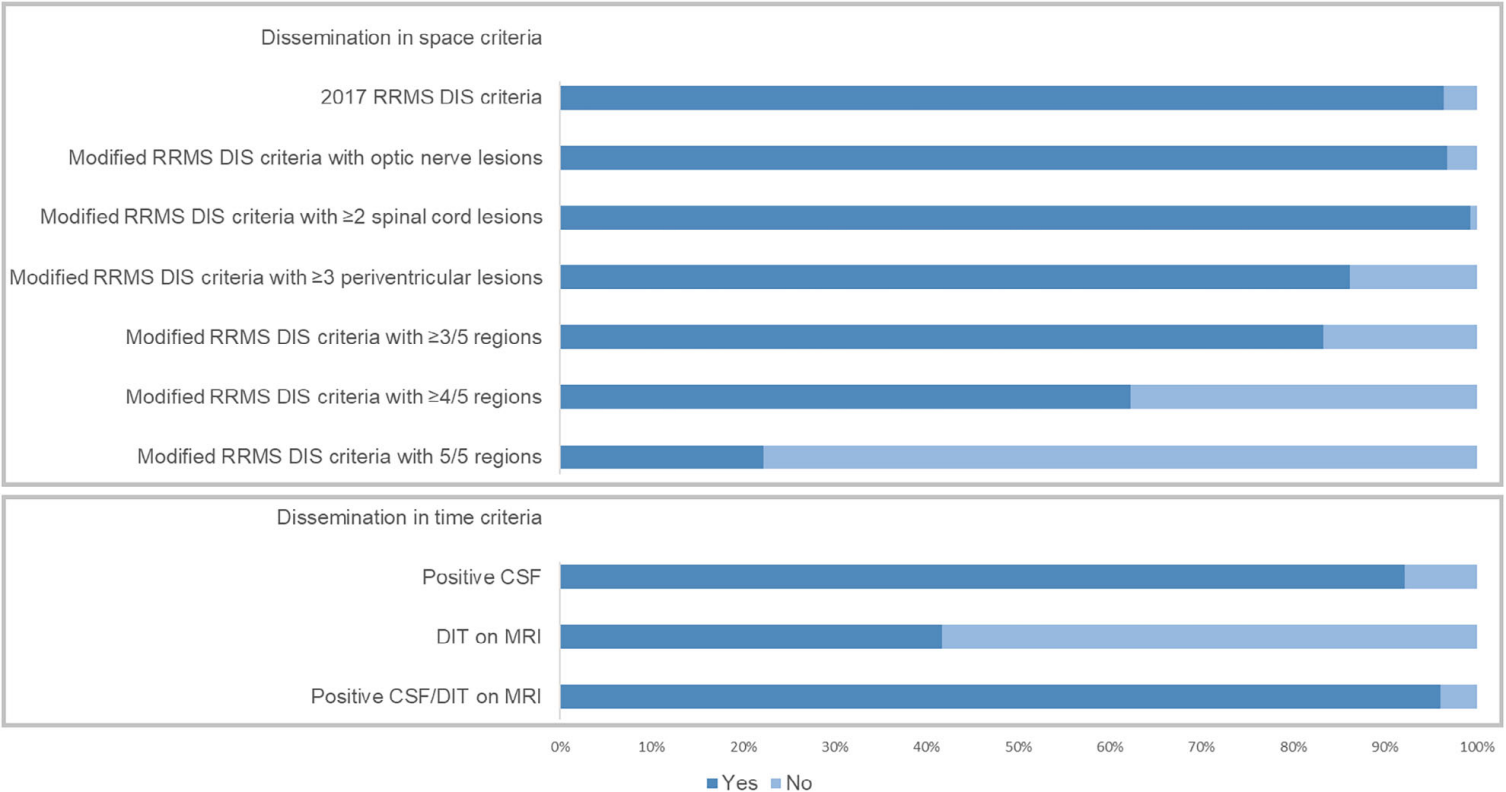
Percentage of primary progressive multiple sclerosis patients fulfilling the 2017 McDonald relapsing–remitting multiple sclerosis (RRMS) diagnostic criteria (dissemination in time [DIS] and dissemination in time [DIT] presented separately), and the modified DIS criteria. MRI = magnetic resonance imaging. [Color figure can be viewed at www.annalsofneurology.org]

The diagnostic performance of the 2017 McDonald RRMS criteria and the modified DIS criteria using the 2017 McDonald PPMS criteria as the gold standard is shown in Tables 2 and 3. Fulfillment of the various criteria is shown in Figure 2.

1. *Performance of 2017 McDonald RRMS diagnostic criteria*

**TABLE 2:** Performance of the McDonald 2017 Relapsing–Remitting Multiple Sclerosis Dissemination in Space Criteria Alone and in Combination with Positive Cerebrospinal Fluid and/or Dissemination in Time Criteria on Magnetic Resonance Imaging

	Sensitivity (95% CI)	Specificity (95% CI)	Accuracy (95% CI)	PPV (95% CI)	NPV (95% CI)
2017 RRMS DIS MRI	96.5% (93.6, 98.2%)	82.5% (67.2, 92.7%)	94.7% (91.7, 96.9%)	97.5% (95.2, 98.7%)	76.7% (63.9, 86.1%)
2017 RRMS DIS MRI plus CSF‐specific OCBs	89.4% (85.2, 92.7%)	95.0% (83.1, 99.4%)	90.1% (86.3, 93.1%)	99.2% (97.0, 99.8%)	55.9% (47.3, 64.2%)
2017 RRMS DIS MRI plus CSF‐specific OCBs or DIT on MRI	92.9% (89.3, 95.6%)	95.0% (83.1, 99.4%)	93.2% (89.8, 95.7%)	99.2% (97.1, 99.8%)	65.5% (55.3, 74.5%)

CI = confidence interval; CSF = cerebrospinal fluid; DIS = dissemination in space; DIT = dissemination in time; MRI = magnetic resonance imaging; NPV = negative predictive value; OCBs = oligoclonal bands; PPV = positive predictive value; RRMS = relapsing–remitting multiple sclerosis.

**TABLE 3:** Performance of the Modified Dissemination in Space Criteria

	Sensitivity (95% CI)	Specificity (95% CI)	Accuracy (95% CI)	PPV (95% CI)	NPV (95% CI)
Modified DIS criteria including the optic nerve(one lesion in ≥2 of 5 locations) alone and in combination with DIT MRI and/or positive CSF					
Modified DIS criteria(≥2 of 5)	96.8% (94.0, 98.5%)	82.5%(67.2, 92.7%)	95.0%(92.1, 97.1%)	97.5%(95.2, 98.7%)	78.6%(65.5, 87.6%)
Modified DIS criteria(≥2 of 5) plus CSF‐specific OCBs	89.7%(85.6–93.0%)	95.0%(83.1–99.4%)	90.4%(86.6–93.4%)	99.2%(97.0–99.8%)	56.7%(48.0–65.1%)
Modified DIS criteria(≥2 of 5) plus CSF‐specific OCBs or DIT on MRI	93.3%(89.7–95.9%)	95.0%(83.1–99.4%)	93.5%(90.2–95.9%)	99.2%(97.2–99.8%)	66.7%(56.3–75.6%)
Modified DIS criteria including ≥2 spinal cord lesions either alone or in combination with 2017 McDonald RRMS DIS criteria and with DIT MRI and/or positive CSF					
≥2 spinal cord lesion as the only DIS criterion	76.6%(71.2–81.4%)	95%(83.1–99.4%)	78.9%(74.0–83.2%)	99.1%(96.5–99.8%)	36.5%(31.5–41.8%)
2017 RRMS DIS or ≥2 spinal cord lesion	99.3%(97.4–99.9%)	77.5%(61.6–89.2%)	96.6%(94.0–98.3%)	96.9%(94.6–98.2%)	93.9%(79.4–98.4%)
2017 RRMS DIS or ≥2 spinal cord lesions plus CSF‐specific OCBs	91.5%(87.6–94.5%)	95.0%(83.1–99.4%)	91.9%(88.4–94.7%)	99.2%(97.1–99.8%)	61.3%(51.8–70.0%)
2017 RRMS DIS or ≥2 spinal cord lesions plus CSF‐specific OCBs or DIT on MRI	95.4%(92.3–97.5%)	95.0%(83.1–99.4%)	95.3%(92.4–97.4%)	99.3%(97.2–99.8%)	74.5%(63.1–83.3%)
Modified criteria requiring ≥3 lesions to confirm involvement of the periventricular region either alone or in combination with 2017 McDonald RRMS DIS criteria and with DIT MRI and/or positive CSF					
Modified DIS criteria requiring ≥3 periventricular lesions	86.2%(81.6–90.0%)	92.5%(79.6–98.4%)	87.0%(82.8–90.4%)	98.8%(96.5–99.6%)	48.7%(41.2–56.3%)
Modified DIS criteria requiring ≥3 periventricular lesions plus CSF‐specific OCBs	80.1%(75.0–84.6%)	97.5%(86.8–99.9%)	82.3%(77.7–86.3%)	99.6%(97.0–99.9%)	41.1%(35.4–47.0%)
Modified DIS criteria requiring ≥3 periventricular lesions plus CSF‐specific OCBs or DIT on MRI	82.3%(77.3–97.8%)	97.5%(86.8–99.9%)	84.2%(79.7–88.0%)	99.6%(97.1–99.9%)	43.8%(37.6–50.2%)

CI = confidence interval; CSF = cerebrospinal fluid; DIS = dissemination in space; DIT = dissemination in time; MRI = magnetic resonance imaging; NPV = negative predictive value; OCBs = oligoclonal bands; PPV = positive predictive value; RRMS = relapsing–remitting multiple sclerosis.

The 2017 McDonald RRMS DIS criteria demonstrated high sensitivity (96.5%), accuracy (94.7%), and PPV (97.5%) for the diagnosis of PPMS, but lower specificity (82.5%). When DIS was combined with CSF‐specific OCBs, specificity was high (95%), but sensitivity (89.4%) and accuracy (90.1%) were lower. Sensitivity (92.9%) and accuracy (93.2%) were slightly higher with the combination of DIS plus positive CSF/DIT‐MRI, and specificity was the same (Table 2).

2 *Including the optic nerve as fifth region in DIS*

One additional patient could be diagnosed with PPMS when the 2017 McDonald RRMS criteria were expanded to include the optic nerve as a fifth DIS region (≥2 of 5 regions). The diagnostic performance was essentially unchanged (Table 3) when compared with the 2017 McDonald RRMS criteria. The results were similar after excluding patients without VEPs (Supplementary Table 3).

3 *Including* ≥*2 spinal cord lesions in DIS*

When the presence of ≥2 spinal cord lesions was used to provide alternative evidence for DIS (ie, patients not fulfilling the 2017 RRMS DIS criteria were tested for the presence of ≥2 spinal cord lesions, including patients with normal brain MRI), 8 additional patients could be diagnosed with PPMS, improving the performance of the DIS criteria: sensitivity (99.3%), accuracy (96.6%), and PPV (96.9%) were high, but the specificity was moderate (77.5); the specificity was higher in combination with positive CSF and/or DIT‐MRI (95%; Table 3).

4 *Periventricular lesion number in DIS criteria*

We investigated the impact of increasing the number of lesions required to confirm involvement of the periventricular region from ≥1 to ≥3, leaving other requirements for DIS unchanged. Modified DIS criteria requiring ≥3 periventricular lesions had moderate sensitivity (82.3%) and accuracy (84.2%), but the specificity (97.5%) and PPV (99.6%) were high for a diagnosis of PPMS (Table 3). In older patients (aged ≥50 years), the sensitivity (87.4% vs 78.9%) and accuracy (89.1% vs 80.4%) were higher than in younger patients (aged <50 years), but the specificity was lower (96.3% vs 100%).

5 *Diagnosis of PPMS on the basis of DIS alone*

We investigated whether PPMS could be diagnosed on the basis of DIS alone in patients with high fulfillment of DIS criteria (lesions in ≥3 regions typically affected in MS), without positive CSF/DIT‐MRI (Table 4). Sensitivity of DIS criteria requiring ≥3 of 4 or 4 of 4 regions was low (55.0% and 34.8%, respectively) for a diagnosis of PPMS, but the specificity (100%) and PPV (100%) were very high. When the optic nerve was included in DIS criteria, the findings were similar; ≥3 of 5, ≥4 of 5, or 5 of 5 regions had reducing sensitivity (83.3%, 62.2%, and 22.2%, respectively), but high specificity (100%).

**TABLE 4:** Performance of the Modified Dissemination in Space Criteria Requiring ≥3 Regions Without Cerebrospinal Fluid ‐Specific Oligoclonal Bands and/or Dissemination in Time Criteria

	Sensitivity (95% CI)	Specificity (95% CI)	Accuracy (95% CI)	PPV (95% CI)	NPV (95% CI)
Modified DIS criteria (one lesion in ≥3 of 4 locations) without DIT MRI or positive CSF					
Modified DIS criteria ≥3 of 4 regions	55.0% (49.0, 60.9%)	100% (91.2, 100%)	60.6% (55.0, 65.9%)	100% (97.7, 100%)	24.0% (21.7, 26.4%)
Modified DIS criteria 4 of 4 regions	34.8% (29.2, 40.6%)	100% (91.2, 100%)	42.9% (37.4, 48.5%)	100% (96.3, 100%)	17.9% (16.6, 19.1%)
Modified DIS criteria including the optic nerve (one lesion in ≥3 of 5 locations) without DIT MRI or positive CSF in patients with VEPs (n = 110)					
Modified DIS criteria ≥3 of 5 regions	83.3% (74.0, 90.4%)	100% (83.2, 100%)	86.4% (78.5, 92.2%)	100% (95.2, 100%)	57.1% (45.7, 67.9%)
Modified DIS criteria ≥4 of 5 regions	62.2% (51.4, 72.2%)	100% (83.2, 100%)	69.1% (59.6, 77.6%)	100% (93.6, 100%)	37.0% (43.4, 64.4%)
Modified DIS criteria 5 of 5 regions	22.2% (14.1, 32.2%)	100% (83.2, 100%)	36.4% (27.4, 46.1%)	100% (83.2, 100%)	22.2% (20.4, 24.2%)

CI = confidence interval; CSF = cerebrospinal fluid; DIS = dissemination in space; DIT = dissemination in time; MRI = magnetic resonance imaging; NPV = negative predictive value; OCBs = oligoclonal bands; PPV = positive predictive value; RRMS = relapsing–remitting multiple sclerosis.

### Findings in Patients Without CSF Examination

When the performance of 2017 RRMS criteria and all the modified DIS criteria above was investigated in all patients (n = 405), even without CSF examination, the performance of each DIS criteria was similar (Supplementary Table 4).

### Diagnostic Algorithm for MS

**FIGURE 3:**
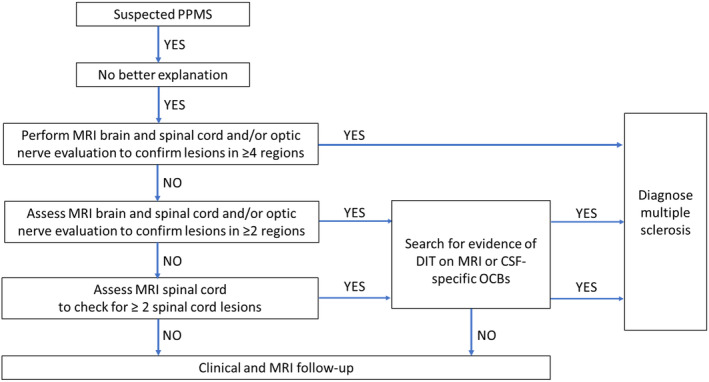
Diagnostic algorithm for suspected primary progressive multiple sclerosis. CSF = cerebrospinal fluid; DIT = dissemination in time; MRI = magnetic resonance imaging; OCBs = oligoclonal bands; PPMS = primary progressive multiple sclerosis. [Color figure can be viewed at www.annalsofneurology.org]

Taking into account the aforementioned findings, we developed an algorithm that presents a new approach to PPMS diagnosis (Figure 3). In patients with suspected PPMS, alternative diagnoses should be excluded. If there are lesions on the brain and spinal MRI (and/or optic nerve) in ≥4 typical regions, MS can be diagnosed. If there are lesions in ≥2 typical regions, or ≥2 spinal cord lesions with normal brain MRI, then positive CSF/DIT‐MRI is required to diagnose MS.

When applying the diagnostic algorithm to the primary cohort (n = 282), after excluding patients with better explanations, 154 (55%) patients had lesions in ≥4 regions using brain and spinal cord MRI, plus VEPs (if available), and could be diagnosed with PPMS without any further diagnostic work‐up. A further 115 (41%) patients DIS in 2 or 3 regions plus positive CSF and/or MRI evidence of DIT, and 8 (3%) additional patients had ≥2 spinal cord lesions plus positive CSF. Application of the algorithm in the cohort used for the primary analysis (all of whom had CSF examination) and the cohort used in the secondary analysis is presented in Supplementary Figure 1.

## Discussion

We showed that application of diagnostic criteria validated in patients with RRMS are similarly suited to diagnosing PPMS. The overall performance of the 2017 McDonald RRMS criteria was high in patients with suspected PPMS, especially when CSF examination was included. Modified RRMS DIS criteria that include the optic nerve as a fifth region appear feasible in PPMS patients, without compromising specificity, and the presence of ≥2 spinal cord lesions may be an alternative way to confirm DIS when brain MRI findings are normal/inconclusive. Among patients with lesions in ≥3 regions typically affected in MS, the specificity for a diagnosis of PPMS was very high, even without positive CSF/DIT‐MRI. Finally, we propose a new diagnostic algorithm applicable to patients with suspected PPMS that should now be tested in relapse‐onset MS.

A single, unified set of criteria for MS has several advantages. Defining MS disease course clinically is challenging^13^; some patients present with a short history of progressive worsening over <12 months (8.2% patients ultimately diagnosed with PPMS had a history of progressive worsening over <12 months at the time of initial evaluation), other patients have multiple relapses with incomplete recovery that can mimic a progressive course, and confirming progression over >12 months may be problematic without objective change, and in patients with comorbidities.^7^, ^15^, ^16^ Misunderstanding of MS diagnostic criteria is common, potentially contributing to both MS misdiagnosis,^17^ and delayed diagnosis.^18^ Separate diagnostic criteria for PPMS adds complexity to MS diagnosis, particularly for neurologists outside of specialist centers. As noted already, a single set of diagnostic criteria not only better reflects MS pathophysiology,^9^ but also simplifies the evaluation of patients with suspected MS, irrespective of the initial clinical symptoms.

Although the 2017 McDonald RRMS criteria perform well for PPMS diagnosis, adding an alternative criterion allowing ≥2 spinal cord lesions enhanced their performance. A very small number of PPMS patients in this cohort (~3%) had normal brain MRI, which is similar to other studies.^19^ These patients otherwise had typical features of PPMS with progressive disease course, multiple spinal cord lesions, and CSF‐specific OCBs. The 2017 McDonald criteria (and earlier iterations) allow for a diagnosis of PPMS in patients with ≥2 spinal cord lesions and CSF‐specific OCBs, reflecting the high value of spinal cord lesions in MS diagnosis and differential diagnosis.^20^ It should be noted that we did not collect data on the topography of spinal cord lesions, or the spinal segments involved, and any 2 spinal cord lesions were considered sufficient for DIS.

In contrast, VEP‐detected optic nerve lesions had little added value, unlike in studies in clinically isolated syndrome (CIS).^21^, ^22^, ^23^ VEPs were abnormal in ~60% of PPMS patients; however, all but 1 patient had sufficient MRI evidence for DIS to make a diagnosis of MS. In a previous MAGNIMS study investigating CIS patients, the percentage of patients with abnormal VEP varied from 19.1% in patients with a non‐optic neuritis presentation, to 76.7% in patients with optic neuritis,^22^ suggesting that the high percentage of PPMS patients with abnormal VEPs reflects a selection bias (ie, VEPs were performed in PPMS with visual symptoms). Importantly, there was no reduction in overall specificity, which is a concern in RRMS patients.^23^, ^24^ The inclusion of the optic nerve lesions as a fifth DIS region in the forthcoming revision of the McDonald diagnostic criteria does not adversely impact PPMS diagnosis. A limitation of this work is the lack of data on orbital MRI and optical coherence tomography (OCT), which can identify optic nerve lesions in patients with suspected MS.^23^ This reflects clinical practice, where orbital MRI and OCT have so far been rarely included in work‐up of suspected PPMS. Future studies should investigate the value of OCT and orbital MRI in patients with suspected PPMS.

PPMS patients typically present at an older age than patients with RRMS.^7^ Older patients (>50 years) had a similar clinical profile to younger patients (<50 years) evaluated for PPMS, with the exception of gadolinium‐enhancing lesions, which were less common, in line with previous studies.^25^ Aging may complicate the application of MRI criteria for DIS and DIT due to white matter lesions related to healthy aging or vascular comorbidities.^8^ There was no central review of MRI in this study to confirm that periventricular, juxtacortical, and infratentorial white matter lesion counts used to apply DIS criteria, or new T2 brain lesions used to apply DIT criteria, were compatible with demyelination rather than non‐specific or vascular lesions. However, all patients were evaluated in specialist MS centers with access to expert neuroradiologists. To address the relatively low specificity of periventricular lesions for MS, a threshold of 3 (rather than 1) periventricular lesions has previously been recommended.^26^ We examined periventricular lesion number for the first time in PPMS patients. Although specificity was high with modified DIS criteria requiring ≥3 periventricular lesions, the sensitivity and accuracy was lower than with criteria requiring ≥1 periventricular lesion, particularly in patients aged <50 years, consistent with previous studies in young adults with CIS.^24^, ^27^, ^28^

RRMS diagnostic criteria require both DIS and DIT,^6^ although CSF‐specific OCBs can substitute for DIT on MRI.^29^, ^30^ PPMS patients present with worsening disability over 12 months (often longer) at the time of initial evaluation, providing clinical evidence already for DIT. However, we found that the combination of DIS and positive CSF/DIT‐MRI had higher specificity for MS diagnosis, in‐keeping with previous studies in RRMS^24^ and PPMS^31^ patients. Positive CSF resulted in higher specificity at the expense of lower sensitivity, although this was partly offset by using DIT on MRI in the small number of patients with negative OCBs (<10% of patients in this cohort). Use of MRI to provide evidence of DIT in PPMS patients does come with some caveats. Gadolinium‐enhancing lesions are less common in PPMS patients, and only one‐third of patients had new T2 brain lesions at follow up. As noted already, new T2 brain lesions are sometimes due to the effects of aging or vascular disease rather than demyelination, which represents a challenge in clinical settings. The relative importance of positive CSF/DIT‐MRI is less in patients with high DIS fulfilment, in whom the probability of MS is higher. We investigated for the first time the diagnostic performance of modified DIS criteria requiring lesions in ≥3 regions alone, without positive CSF/DIT‐MRI. Higher fulfillment of DIS criteria had very high specificity and could be used to “rule‐in” MS (in our algorithm we suggest DIS in ≥4 regions to be conservative), with lumbar puncture reserved for patients with DIS in ≤3 regions or patients with multiple spinal cord lesions. Studies are underway to investigate whether higher fulfillment of DIS criteria in patients with suspected RRMS is useful to diagnose MS in cases with relapse onset disease.

Testing the performance of diagnostic criteria for PPMS is not straight‐forward. In CIS patients, a second clinical attack has been adopted as the gold standard by which diagnostic criteria performance is judged, at least historically.^24^ No such gold standard exists for PPMS diagnosis. We used the 2017 McDonald criteria as the gold standard, reflecting the latest set of recommendations for clinicians diagnosing MS,^6^ whereas previous studies used either historical criteria^31^ or best clinician judgement.^32^ In the present study, patients were evaluated for suspected PPMS, but ultimately diagnosed with other disorders, were used as a control group. The 2017 McDonald criteria recommend exclusion of other disorders,^6^ but we considered the inclusion of PPMS mimics to be a necessary step to calculate specificity. This novel approach has resulted is the largest study to date investigating PPMS diagnostic criteria, which have significantly lagged behind evolution of RRMS criteria.

The present study had some limitations, in addition to the lack of orbital MRI and OCT data. We collected information on patients seen at 5 specialist centers with evaluation as part of routine clinical care. The high prevalence (or pretest probability) of PPMS in the studied cohort may be interpreted as limiting generalizability of the results, as this may have led to a very high PPV; however, the prevalence of PPMS is not expected to influence specificity, sensitivity, and accuracy of the criteria, and the 2017 McDonald criteria are not intended for application in unselected patients. Importantly, data on ethnicity and comorbidities were not collected as part of the current study, and future studies should evaluate the performance of PPMS diagnostic criteria in these special patient populations. Standardized research protocols were not used for data acquisition and there was no central MRI review, unlike in previous studies.^24^, ^33^ However, a criticism of such studies is that the findings may not be generalizable to real‐world settings. Inherent to a study of this nature is missing data. Approximately 20% of patients did not undergo lumbar puncture, and our primary analysis only included patients with CSF examination. However, we repeated all analyses in the larger cohort of patients (which included those without CSF examination), to address any concern about selection bias, confirming similar findings. Although not mandatory, CSF examination is currently recommended as part of the work‐up of patients with suspected PPMS,^6^, ^7^, ^8^ because PPMS patients are less likely to have new or gadolinium‐enhancing lesions on MRI^34^ (thus less likely to fulfill MRI criteria for DIT), and OCB status was felt to be able to substitute for DIT‐MRI when applying the 2017 McDonald RRMS criteria. We did not evaluate emerging diagnostic biomarkers for MS, such as the kappa free light chain index,^35^ central vein sign,^36^ or paramagnetic rim lesions,^37^ which have primarily been tested in RRMS patients. These diagnostic biomarkers are not currently widely available in clinical settings, and were not collected as part of the current retrospective study. Finally, although our aim was to a develop a single, unified set of diagnostic criteria for MS, we only evaluated patients with suspected PPMS. The 2017 McDonald criteria have already been widely validated in CIS patients,^33^, ^38^ and our proposed diagnostic algorithm now needs to be tested in a relapsing MS population.

## Conclusions

The diagnostic criteria for RRMS have high accuracy when applied to patients under investigation for suspected PPMS, representing a simplified approach to MS diagnosis, irrespective of whether the clinical presentation is with a relapse or progressive worsening. As RRMS diagnostic criteria evolve to include optic nerve lesions and multiple spinal cord lesions, or requirements for DIT are relaxed in patients with high DIS fulfillment, these changes are also feasible for PPMS without compromising diagnostic performance. The application of the diagnostic algorithm, here proposed for patients with progressive disease at onset, to patients with relapse‐onset MS, will be investigated in future studies. Overall, the present findings provide a strong rationale for a single unified set of diagnostic criteria for MS. Future studies can build on these findings and incorporate novel diagnostic biomarkers, such as central vein sign and paramagnetic rim lesions.

## Author Contributions

W.J.B., A.J.T., A.T.T., and O.C. contributed to conception and design of the study; W.J.B., A.V.J., M.S., E.S., J.K., L.B., F.B., A.R., J.S.G., M.T., M.A.R., F.E., M.A., M.F., B.B., A.L., B.S., and X.M. contributed to the acquisition and analysis of data. All authors contributed to drafting the text or preparing the figures.

## Potential Conflicts of Interest

J.K., F.B., A.R., J.S.G., M.T., M.A.R., M.F., BS, XM, AJT, and OC participated in the 2023 McDonald Diagnostic Criteria Review Meeting in November/December 2023, where data presented in this manuscript were presented and discussed. The conception, design, and analysis of this study, and the preparation of this manuscript are independent of the 2023 McDonald Diagnostic Criteria Review Meeting. The authors have nothing to report.

## Supporting information


Data S1.

## Data Availability

The corresponding author has full access to all of the data in the study, and takes responsibility for the data integrity and data analysis. The anonymized dataset analyzed in this study is available from the corresponding author on reasonable request.
