# Supplementary material for: Towards a Unified Set of Diagnostic Criteria for Multiple Sclerosis
Source: Ann Neurol. 2024 Nov 28;97(3):571–82. doi: 10.1002/ana.27145 (PMC11831880; doi:10.1002/ana.27145)
Supplement: Supplementary file 1 — Data S1. [file ANA-97-571-s001.docx]

**Supplementary Table 1. Baseline characteristics of patients with PPMS grouped by age at the time of initial evaluation**

|  | **Age <50 years**  **(n=158)** | **Age >50 years**  **(n=124)** |
| --- | --- | --- |
| **Age, mean (SD), years** | 41.6 (7.6) | 55.8 (9.9) |
| **Female, n (%)** | 66 (41.7) | 67 (54.0) |
| **Clinical presentation, n (%)**  Myelopathy  Brainstem/cerebellar syndrome  Other presentations ^a^ | 136 (86.0)  14(8.8)  9 (5.7) | 110 (88.7)  8 (6.5)  5 (4.0) |
| **Progression over >12 months, n (%)** | 155 (98.1) | 104 (83.9) |
| **Brain MRI findings, n (%)**  Normal ^b^  ≥1 Periventricular lesions  ≥1 Cortical/juxtacortical lesions  ≥1 Infratentorial lesions  ≥1 Gadolinium-enhancing lesions at baseline ^c^  ≥1 New lesion at follow-up ^d^ | 6 (3.8)  144 (91.1)  120 (76.0)  104 (65.8)  19/115 (16.5)  48/130 (36.9) | 2 (1.6)  117 (94.4)  82 (66.1)  74 (59.7)  5/82 (6.1)  25/91 (27.5) |
| **Spinal cord MRI findings, n (%)**  Normal  1 Spinal cord lesion  ≥2 Spinal cord lesions  ≥1 Gadolinium-enhancing lesions at baseline ^c^  ≥1 New lesion at follow-up ^d^ | 12 (7.6)  25 (15.8)  121 (76.6)  11/95 (11.6)  17/81 (21.0) | 8 (6.5)  21 (16.9)  95 (76.6)  3/61 (4.9)  14/52 (21.2) |
| **CSF-specific oligoclonal bands, n (%)** | 145 (91.8) | 115 (92.7) |
| **Abnormal VEPs, n (%),** ^e^ | 36/57 (63.2) | 17/33 (51.5) |

^a^ Other presentations for example progressive hemiparesis, multifocal symptoms

^b^ Patients without T2-hyperintense lesions in the periventricular, cortical/juxtacortical or infratentorial regions

^c^ Over the number of patients who had a post-contrast T1 scan

^d^ Over the number of patients who had a follow-up MRI scan

^e^ Over the number of patients with VEP information

**Supplementary Table 2. Alternative diagnoses in patients evaluated for suspected PPMS with a CSF examination**

|  | Frequency, n |
| --- | --- |
| Cervical spondylotic myelopathy | 9 |
| Inflammatory myelopathies  Solitary sclerosis  Atypical transverse myelitis  Post-infectious myelitis  Neurosarcoidosis | 9  5  2  1  1 |
| Motor neurone disease  Primary lateral sclerosis  Amyotrophic lateral sclerosis | 5  3  2 |
| Functional neurological disorder | 3 |
| Degenerative parkinsonism | 3 |
| Idiopathic cerebellar ataxia | 2 |
| Paraneoplastic syndromes | 2 |
| Small vessel cerebrovascular disease | 1 |
| Normal pressure hydrocephalus | 1 |
| Superficial siderosis | 1 |
| Genetic cerebellar ataxia | 1 |
| CANVAS | 1 |
| HIV infection | 1 |
| B12 deficiency | 1 |

Abbreviations: CANVAS = cerebellar ataxia with neuropathy and vestibular areflexia syndrome

**Supplementary Table 3. Performance of the McDonald 2017 RRMS criteria and modified RRMS criteria including optic nerve topography in 110 patients with visual evoked potentials**

|  | **Sensitivity**  **(95%CI)** | **Specificity**  **(95%CI)** | **Accuracy**  **(95%CI)** | **PPV**  **(95% CI)** | **NPV**  **(95% CI)** |
| --- | --- | --- | --- | --- | --- |
| ***RRMS 2017 DIS criteria*** | | | | | |
| **RRMS 2017 DIS** | 93.3%  (86.1% - 97.5%) | 80.0%  (56.3 – 94.3%) | 90.9%  (83.9 - 95.6%) | 95.5%  (89.7 - 98.1%) | 72.7%  (54.4 to 85.6%) |
| **RRMS 2017 DIS plus CSF-specific oligoclonal bands** | 92.2%  (84.6% - 96.8%) | 95.0%  (75.1 – 99.9%) | 92.7%  (86.2 – 96.8%) | 98.8%  (92.5 - 99.8%) | 73.1%  (57.0 – 84.8%) |
| **RRMS 2017 DIS plus CSF-specific oligoclonal bands/DIT** | 93.3%  (86.1 - 97.5%) | 95.0%  (75.1 - 99.9%) | 93.6%  (87.3 - 97.4%) | 98.8%  (92.6 - 99.8%) | 76.0%  (59.2 - 87.4%) |
| ***Modified RRMS 2017 DIS criteria with addition of optic nerve lesions*** | | | | | |
| **RRMS 2017 DIS-ON** | 94.4%  (87.5 -98.2%) | 80.0%  (56.3% - 94.3%) | 91.8%  (85.0 – 96.2%) | 95.5%  (89.8 98.1%) | 76.2%  (57.0 - 88.5%) |
| **RRMS 2017 DIS-ON plus CSF-specific oligoclonal bands/DIT** | 93.3%  (86.1 – 97.5%) | 95.0%  (75.1 - 99.9%) | 93.6%  (87.3 - 97.4%) | 98.8%  (92.6 - 99.8%) | 76.0%  (59.2 - 87.4%) |
| **RRMS 2017 DIS-ON plus CSF-specific oligoclonal bands/DIT** | 94.4%  (87.5 - 98.2%) | 95.0%  (75.1 – 99.9%) | 94.5%  (88.5 - 98.0%) | 98.8%  (92.6 - 99.8%) | 79.2%  (61.7 – 90.0%) |

Abbreviations: CI=confidence interval, DIS=dissemination in space, DIT=dissemination in time, NPV=negative predictive value, PPV=positive predictive value

**Supplementary Table 4. Performance of 2017 McDonald 2017 RRMS and all the modified DIS criteria alone and in combination with positive CSF and/or DIT criteria when 405 patients (358 with McDonald PPMS and 47 with alternative diagnoses) with a CSF examination and/or follow-up MRI.**

|  | **Sensitivity**  **(95%CI)** | **Specificity**  **(95%CI)** | **Accuracy**  **(95%CI)** | **PPV**  **(95% CI)** | **NPV**  **(95% CI)** |
| --- | --- | --- | --- | --- | --- |
| ***2017 RRMS DIS and DIT criteria*** | | | | | |
| **2017 RRMS DIS MRI** | 97.2%  (94.9 – 98.7%) | 83.0%  (69.2 – 92.3%) | 95.6%  (93.1 – 97.3%) | 97.8%  (95.9 – 98.8%) | 79.6%  (67.6 – 87.9%) |
| **2017 RRMS DIS MRI plus CSF-specific OCBs** | 71.0%  (66.0 – 75.6%) | 95.7%  (85.5 – 99.5%) | 73.8%  (69.3 – 78.0%) | 99.2%  (97.0 – 99.8%) | 30.2%  (26.7 – 34.0%) |
| **2017 RRMS DIS MRI plus CSF-specific OCBs or DIT on MRI** | 86.0%  (82.0 – 89.5%) | 95.7%  (85.5 – 99.5%) | 87.2%  (83.5 – 90.3%) | 99.4%  (97.5 – 99.8%) | 47.4%  (40.9 – 54.0%) |
| ***Modified DIS criteria including the optic nerve (one lesion in ≥2 out of 5 locations) alone and in combination with DIT MRI or positive CSF*** | | | | | |
| **Modified DIS criteria (≥2/5)** | 97.5%  (95.3 – 99.5%) | 95.7%  (85.5 – 99.5%) | 97.3%  (95.2 – 98.6%) | 99.4%  (97.8 – 99.9%) | 83.3%  (72.3 – 90.5%) |
| **Modified DIS criteria (≥2/5) plus CSF-specific OCBs** | 71.2%  (66.2 – 75.9%) | 95.7%  (85.5 – 99.5%) | 74.1%  (69.5 – 78.3%) | 99.2%  (97.0 – 99.8%) | 30.4%  (26.9 – 34.2%) |
| **Modified DIS criteria (≥2/5) plus CSF-specific OCBs or DIT on MRI** | 86.3%  (82.3 – 89.7%) | 95.7%  (85.5 – 99.5%) | 87.4%  (83.8 – 90.5%) | 99.4%  (97.6 – 99.8%) | 47.9%  (41.3 – 54.5%) |
| ***Modified DIS criteria including at least two spinal cord lesions either alone or in combination with RRMS DIS criteria and with DIT MRI or positive CSF*** | | | | | |
| **≥2 spinal cord lesion as the only DIS criterion** | 80.2%  (75.7 – 84.2%) | 95.7%  (85.5 – 99.5%) | 82.0%  (77.9 – 85.6%) | 99.3%  (97.4 – 99.8%) | 38.8%  (33.8 – 44.1%) |
| **2017 RRMS DIS or ≥2 spinal cord lesion** | 99.4%  (98.0 – 99.9%) | 78.7%  (64.3 – 89.3%) | 97.0%  (94.9 – 98.5%) | 97.3%  (95.4 – 98.4%) | 94.9%  (82.2 – 98.7% |
| **2017 RRMS DIS or ≥2 spinal cord lesions plus CSF-specific OCBs** | 72.6%  (67.7 – 77.2%) | 95.7%  (85.5 – 99.5%) | 75.3%  (70.8 – 79.4%) | 99.2%  (97.1 – 99.8%) | 31.5%  (27.7 – 35.5%) |
| **2017 RRMS DIS or ≥2 spinal cord lesions plus CSF-specific OCBs or DIT on MRI** | 86.0%  (82.0 – 89.5%) | 95.7%  (85.5 – 99.5%) | 87.2%  (83.5 – 90.3%) | 99.4%  (97.5%) | 47.4%  (40.9 – 54.0%) |
| ***Modified criteria requiring* ≥3 *lesions to confirm involvement of the periventricular region either alone or in combination with 2017 McDonald RRMS DIS criteria and with DIT MRI and/or positive CSF*** | | | | | |
| **Modified DIS criteria requiring** **≥3 periventricular lesions** | 86.6%  (82.6 – 90.0%) | 93.6%  (82.5 – 98.7%) | 87.4%  (83.8 – 90.5%) | 99.0%  (97.2 – 99.7%) | 47.8%  (41.1 – 54.7%) |
| **Modified DIS criteria requiring** **≥3 periventricular lesions plus CSF-specific OCBs** | 63.4%  (58.2 – 68.4%) | 97.9%  (88.7 – 100%) | 67.4%  (62.6 – 72.0%) | 88.4%  (84.9 – 91.4%) | 26.0%  (23.3 – 28.3%) |
| **Modified DIS criteria requiring** **≥3 periventricular lesions plus CSF-specific OCBs or DIT on MRI** | 76.0%  (71.2 – 80.3%) | 97.9%  (88.7 – 100%) | 78.5%  (74.2 – 82.4%) | 99.6%  (97.5 – 100%) | 34.9%  (30.7 – 39.3%) |
| ***Modified DIS criteria (one lesion in ≥3 out of 4 locations) without DIT MRI or positive CSF*** | | | | | |
| **Modified DIS criteria** ≥**3/4 regions** | 79.9%  (75.4, 83.9%) | 100%  (92.5, 100%) | 82.2%  (78.1, 85.8%) | 100%  (98.7, 100%) | 39.5%  (34.7, 44.5%) |
| **Modified DIS criteria 4/4 regions** | 48.6%  (43.3, 53.9%) | 100%  (92.5, 100%) | 54.6%  (49.6, 59.5%) | 100%  (97.9, 99.8%) | 20.3%  (18.8, 22.0%) |
| ***Modified DIS criteria including the optic nerve (one lesion in ≥3 out of 5 locations) without DIT MRI or positive CSF in patients with VEPs (n=142)*** | | | | | |
| **Modified DIS criteria** ≥**3/5 regions** | 84.3%  (77.1, 90.0%) | 91.7%  (61.5, 99.8%) | 84.9%  (78.1, 90.3%) | 99.1%  (94.5, 99.9%) | 33.3%  (25.5, 44.6%) |
| **Modified DIS criteria** ≥**4/5 regions** | 64.9%  (56.2, 73.0%) | 100%  (73.5, 100%) | 67.8%  (59.6, 75.3%) | 100%  (95.9, 100%) | 20.3%  (16.9, 24.3%) |
| **Modified DIS criteria** 5**/5 regions** | 29.9%  (22.3, 38.4%) | 100%  (73.5, 100%) | 35.6%  (27.9, 44.0%) | 100%  (91.2, 100%) | 11.3%  (10.3, 12.5%) |

Abbreviations: CI=confidence interval, DIS=dissemination in space, DIT=dissemination in time, NPV=negative predictive value, PPV=positive predictive value

**Supplementary Figure 1. Application of the proposed diagnostic algorithm in patients included in (A) the primary analysis (n=282) and (B) the secondary analysis (n=358)**

**A**

**
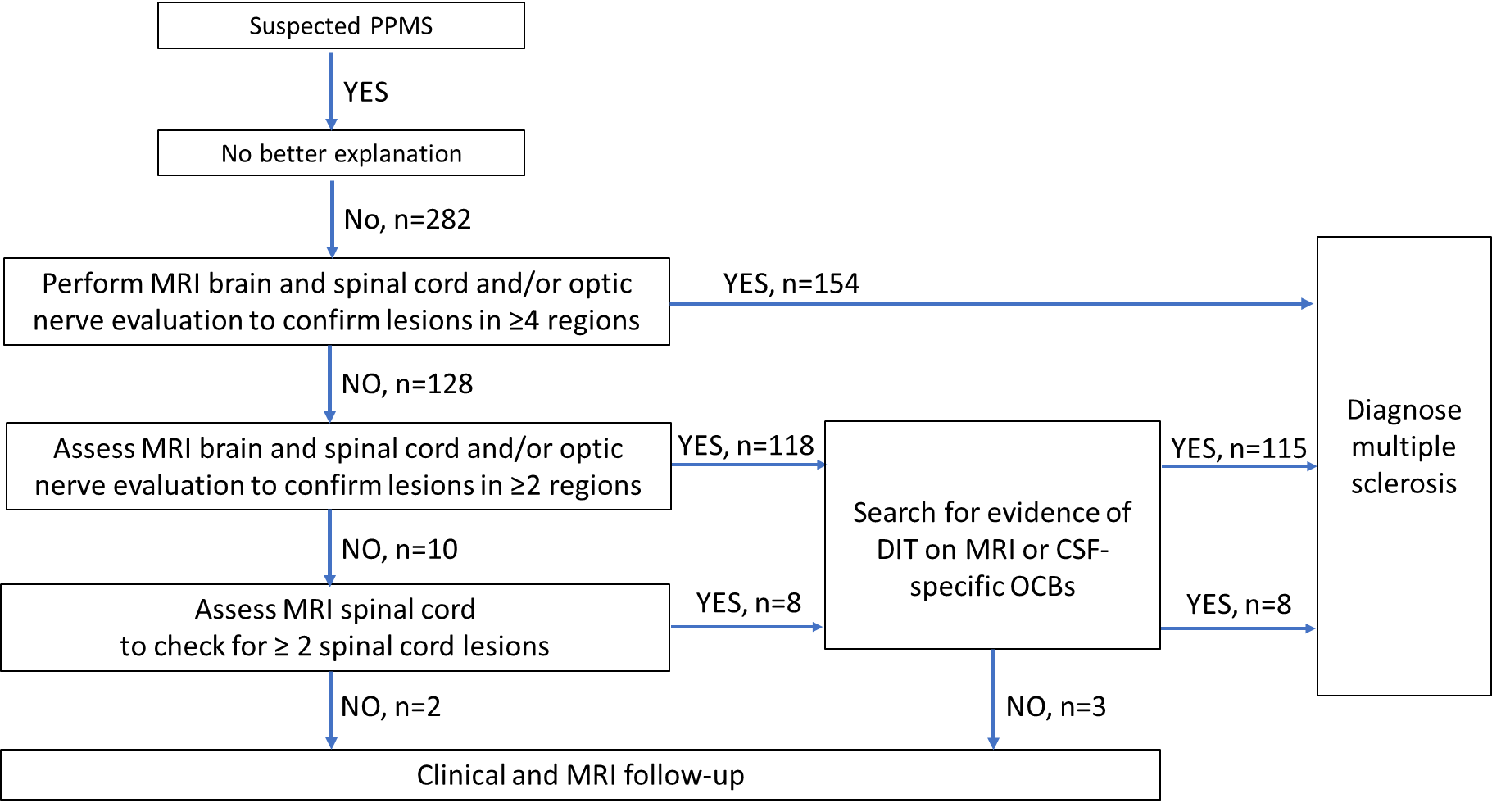
**

**B**

**
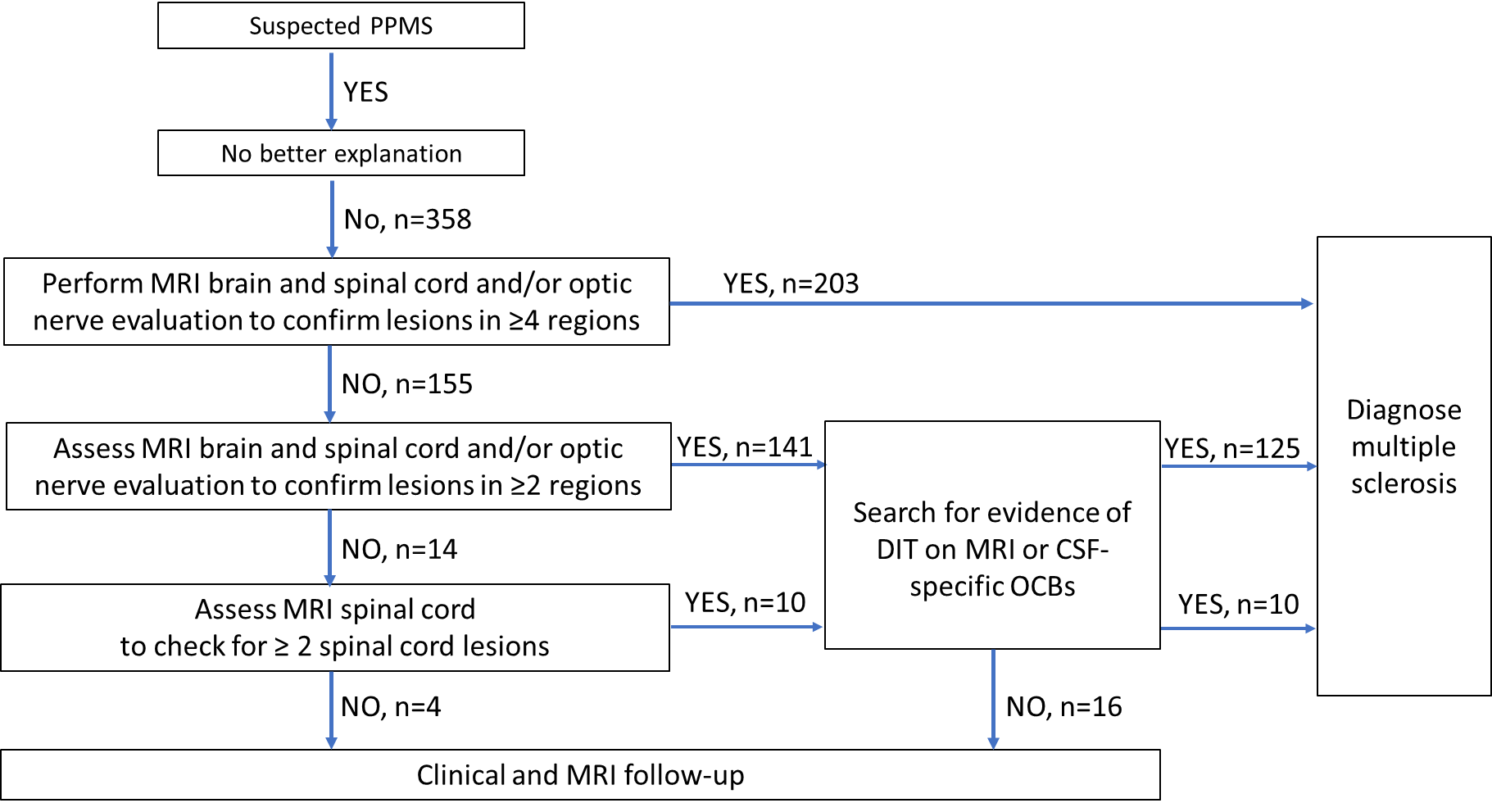
**
